# Random mutations and job opportunities

**DOI:** 10.3325/cmj.2022.63.310

**Published:** 2022-06

**Authors:** Charles H. Calisher

**What a mess!** Clusters of COVID-19 cases here, outbreaks there, catastrophic epidemics somewhere else, and all the while asymptomatic individuals (not “asymptomatic cases” because people who are classified as “cases” always, or should, have symptoms) allowing the virus (SARS-CoV-2) to spread silently between unsuspecting individuals. COVID-19 (it is now 2022, so perhaps “COVID-19-22”, or better, “COVID”?) is racing around the world and then racing around the world again. Vaccines have been produced and most are effective in most people. The number of reported cases and deaths in Africa are very low but we do not know why. We have evidence that anthroponotic transmission (human to non-human vertebrate) is known to occur in chimpanzees, gorillas, giant anteaters, bottlenose dolphins, horses, alligators, bats (not likely to be transmitted to bats from humans), sheep, pangolins, house cats, tigers, lions, minks, deer, and other forms of wildlife and domestic vertebrates. Experimental infections conducted by several research groups have shown that monkeys, hamsters, ferrets, cats, dogs, pigs, tree shrews, various rodents, and fruit bats are permissive, some of them only experimentally, while poultry seem to be resistant (1,2). That mink (*Neovison vison*) and white-tailed deer (*Odocoileus virginianus*) may also serve as reservoirs of SARS-CoV-2 compounds the devastating problems caused by asymptomatic infections of some humans. If these animals infected with a strain, or strains, of SARS-CoV-2 that infects humans, can produce sufficient loads of virus to infect humans, and now may serve as reservoirs of the virus, we will not rid ourselves of it unless and until they are vaccinated or otherwise immunized against it, an unlikely scenario. Other wild vertebrates may also cause the same problems.

As long as there is a single person who is not immune to SARS-CoV-2, which causes COVID, there is a possibility for human infection by the virus and then mutation of that virus, and then of course there are all the susceptible newborns to follow. People who are not immune to variants of the virus may serve as its reservoirs but, worse, replication of the virus sooner or later results in production of mutants that are somewhere between slightly different to astonishingly different from the parental (original) virus, whatever that was. If all humans and other vertebrates were infected and immune to this virus the process would end. That is unlikely to occur, so we now race to prevent random, unpredicted mutations. But how can we predict the unpredictable?

The bad news is that we await the time when SARS-CoV-2 becomes less pathogenic and more endemic. One piece of good news for some, particularly students, is that all this unknowing provides job opportunities for young people interested in virology, in vaccine manufacturing, in viral ecology, and in much more. Henry David Thoreau said, “People talk about Bible miracles because there is no miracle in their lives. Cease to gnaw the crust. There is ripe fruit over your head.” This epidemic is an example of such “ripe fruit;” the prevention of or response to the occurrence of random mutations. Thus far, there is no evidence that we can prevent anything that is random, so how might we begin suppressing random mutations? Burke (3) has speculated excellently on possible evolutionary scenarios for this virus. Perhaps a pan-coronavirus vaccine can be created. Perhaps for every one dose of vaccine that is refused, 10 or 1000 doses of currently available vaccines might be donated to countries that are underserved. Perhaps from a great many ideas, one will be useful. (Linus Pauling: “The best way to get a good idea is to get lots of ideas.”)

**What is the origin of SARS-CoV-2?** This is the focus of political and virological efforts and arguments. The answer may matter politically but not virologically. We have survived nicely without knowing the definitive origin of yellow fever virus, of tick-borne encephalitis virus(es), of hepatitis A virus, of smallpox virus. These are questions the answers to which would build very nice careers for the person or people who find them but, at this point, few care and, really, at this late date, it does not matter epidemiologically.

Students, or others who want to change careers, might find that the current agonies offer a rare opportunity. Jobs now in existence but declining as opportunity: coal miner, taxi driver, surveyor or mapping technicians, print media, herder, travel agent, even diagnosticians; these occupations are or will soon be replaced by automated machinery (artificial intelligence) and the like.

Surely many governmental and non-governmental organizations soon will awaken and be pouring funds into even more research regarding virus evolution and ecology. There being no real rush, it might even be a good idea if students would take an extra year of pre-graduation studies to become more informed about the humanities, geology, psychology, writing, Great Books readings, logic, etymology, cooking, accounting and personal finance, world history, physical education, political science, statistics, gardening, or even taxonomy. These would provide opportunities for the student to become a well-rounded person, one with an education, rather than one who is trained. As importantly, graduate work in virology, evolution, ecology, and other specialties would prepare one for a well-paying job and opportunities to travel to places you have never wanted to visit and to eat food you never wanted to eat.

It will cost a large fortune to rebuild Ukraine and part of the funds surely will go to rebuilding schools, laboratories, places for administrators to sit, and grocery stores, as well as libraries, restaurants, and petrol stations. Of course, if all else fails, everyone is welcome to come to the USA, arguably the largest lunatic asylum in the world. Start preparing by listening to the Eagles: *https://www.youtube.com/watch?v=FVsbvFkhzY4.*

## References

[1] Abdel-Moneim AS Abdelwhab EM Evidence for SARS-CoV-2 Infection of Animal Hosts. Pathogens 2020 9 529 10.3390/pathogens9070529 32629960 PMC7400078

[2] Centers for Disease Control and Prevention. Animals and COVID-19. Available from: https://www.cdc.gov/coronavirus/2019-ncov/daily-life-coping/animals.html*.* Accessed: May 25, 2022.

[3] Burke DS. Coronaviruses are ‘clever’: Evolutionary scenarios for the future of SARS-CoV-2. Available from: https://www.statnews.com/2022/02/16/coronaviruses-are-clever-evolutionary-scenarios-for-the-future-of-sars-cov-2/. Accessed: May 25, 2022.

